# rDNA Chromatin Activity Status as a Biomarker of Sensitivity to the RNA Polymerase I Transcription Inhibitor CX-5461

**DOI:** 10.3389/fcell.2020.00568

**Published:** 2020-07-03

**Authors:** Jinbae Son, Katherine M. Hannan, Gretchen Poortinga, Nadine Hein, Donald P. Cameron, Austen R. D. Ganley, Karen E. Sheppard, Richard B. Pearson, Ross D. Hannan, Elaine Sanij

**Affiliations:** ^1^Peter MacCallum Cancer Centre, Melbourne, VIC, Australia; ^2^Sir Peter MacCallum Department of Oncology, The University of Melbourne, Parkville, VIC, Australia; ^3^ACRF Department of Cancer Biology and Therapeutics, The John Curtin School of Medical Research, Australian National University, Canberra, ACT, Australia; ^4^Department of Biochemistry and Molecular Biology, The University of Melbourne, Parkville, VIC, Australia; ^5^Department of Medicine, St. Vincent’s Hospital, The University of Melbourne, Parkville, VIC, Australia; ^6^School of Biological Sciences, The University of Auckland, Auckland, New Zealand; ^7^Department of Biochemistry and Molecular Biology, Monash University, Clayton, VIC, Australia; ^8^School of Biomedical Sciences, The University of Queensland, Brisbane, QLD, Australia; ^9^Department of Clinical Pathology, The University of Melbourne, Parkville, VIC, Australia

**Keywords:** RNA polymerase I, CX-5461, ovarian cancer, DNA damage response, rDNA copy number

## Abstract

Hyperactivation of RNA polymerase I (Pol I) transcription of ribosomal RNA (rRNA) genes (rDNA) is a key determinant of growth and proliferation and a consistent feature of cancer cells. We have demonstrated that inhibition of rDNA transcription by the Pol I transcription inhibitor CX-5461 selectively kills tumor cells *in vivo*. Moreover, the first-in human trial of CX-5461 has demonstrated CX-5461 is well-tolerated in patients and has single-agent anti-tumor activity in hematologic malignancies. However, the mechanisms underlying tumor cell sensitivity to CX-5461 remain unclear. Understanding these mechanisms is crucial for the development of predictive biomarkers of response that can be utilized for stratifying patients who may benefit from CX-5461. The rDNA repeats exist in four different and dynamic chromatin states: inactive rDNA can be either methylated silent or unmethylated pseudo-silent; while active rDNA repeats are described as either transcriptionally competent but non-transcribed or actively transcribed, depending on the level of rDNA promoter methylation, loading of the essential rDNA chromatin remodeler UBF and histone marks status. In addition, the number of rDNA repeats per human cell can reach hundreds of copies. Here, we tested the hypothesis that the number and/or chromatin status of the rDNA repeats, is a critical determinant of tumor cell sensitivity to Pol I therapy. We systematically examined a panel of ovarian cancer (OVCA) cell lines to identify rDNA chromatin associated biomarkers that might predict sensitivity to CX-5461. We demonstrated that an increased proportion of active to inactive rDNA repeats, independent of rDNA copy number, determines OVCA cell line sensitivity to CX-5461. Further, using zinc finger nuclease genome editing we identified that reducing rDNA copy number leads to an increase in the proportion of active rDNA repeats and confers sensitivity to CX-5461 but also induces genome-wide instability and sensitivity to DNA damage. We propose that the proportion of active to inactive rDNA repeats may serve as a biomarker to identify cancer patients who will benefit from CX-5461 therapy in future clinical trials. The data also reinforces the notion that rDNA instability is a threat to genomic integrity and cellular homeostasis.

## Introduction

**FIGURE 1:**
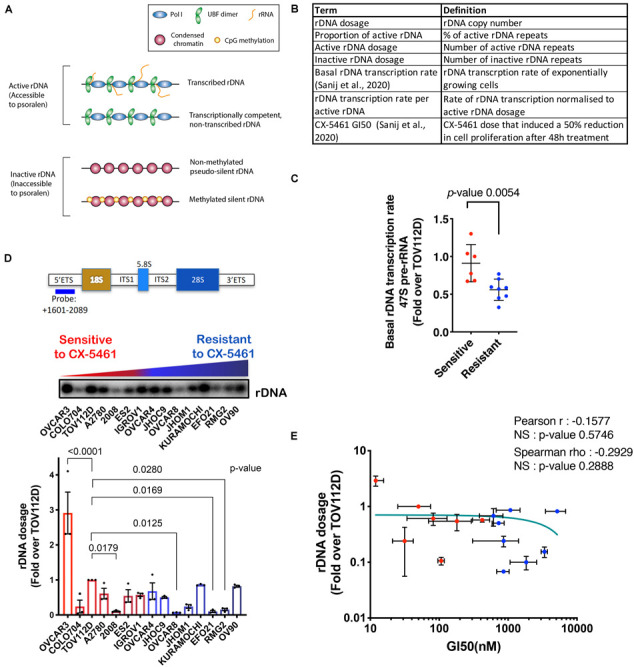
**(A)** Schematic of the rDNA chromatin states. **(B)** A summary of the rDNA activity parameters assessed in this study. **(C)** Basal rDNA transcription rates of OVCA cell lines was reported in Sanij et al. (2020). We have re-used the data under the Creative Commons Attribution 4.0 International License [http://creativecommons.org/licenses/by/4.0/]. 47S rRNA levels were determined in exponentially growing OVCA cell lines by qRT-PCR analysis using the ETS2 primers (Supplementary Table S3) specific to the external transcribed spacer 5’ETS. Expression levels in each cell line were normalized to Vimentin mRNA and expressed as fold change relative to TOV112D cells. Each dot represents the mean value of *n* = 3 biologically independent experiments per cell line (Individual data points are provided in Sanij et al., 2020). Error bars represent mean ± SD. Statistical analysis was performed using a two-tailed unpaired *t* test. **(D)** OVCA cell lines display a range of rDNA content. Top panel, a schematic of the transcribed region of the rDNA repeat detailing the position of the probe used for Southern blotting and a representative Southern blot of gDNA from 15 OVCA cell lines, ordered by increasing GI50 values of CX-5461 as reported in Sanij et al. (2020). gDNA was isolated from 10^6^ cells and Southern blotting for rDNA performed and quantitated using ImageQuant pTL software (GE Healthcare). Graph represents rDNA dosage expressed as fold over that for TOV112D; *n* = 3; mean ± SEM. CX-5461-sensitive cell lines are indicated in red and resistant cell lines are in blue. Statistical analysis was performed using two-sided one-way ANOVA Dunnett’s multiple comparisons test. **(E)** Correlation analysis of rDNA dosage and sensitivity to CX-5461 (GI50) in 15 OVCA cell lines using GraphPad Prism. Error bars represent mean ± SD. Pearson’s r is -0.16, NS (not significant) denotes *p*-value > 0.05; Spearman’s rho is −0.29, NS (not significant) denotes *p*-value > 0.05.

Transcription of the rDNA repeats by Pol I within the nucleoli is a critical step in ribosome biogenesis and accounts for over 60% of all cellular transcription (Warner, 1999; Moss and Stefanovsky, 2002; Moss et al., 2007). The rDNA encodes the 47S pre-rRNA precursor of the 18S, 5.8S, and 28S rRNAs, which together with 5S rRNA constitute the RNA component of ribosomes. The rDNA is organized into large blocks of tandem repeats, with 400–600 repeats divided among the five pairs of acrocentric chromosomes in the human genome (Stults et al., 2009). Modulation of transcriptional rates can be achieved by regulating Pol I transcription initiation, elongation, and/or processing of the 47S rRNA precursor (Stefanovsky et al., 2001; Schneider, 2012; Goodfellow and Zomerdijk, 2013; Hung et al., 2017). Remarkably, only a fraction of the rDNA repeats are actively transcribed at any one time, providing an additional layer of regulation with transcription output being determined at two levels: the active copy number in combination with the rate of transcription per rDNA repeat (Sanij et al., 2008; Zentner et al., 2011; Hamdane et al., 2014). However, the majority of short-term regulation affects rDNA transcription rate through changing the rate of transcription from active genes, reviewed in McStay and Grummt (2008), Sanij and Hannan (2009), Goodfellow and Zomerdijk (2013). In mammalian cells, the rDNA chromatin can exist in active or inactive states (Figure 1A) [reviewed in Sanij and Hannan (2009), Hamperl et al. (2013), Poortinga et al. (2014), Moss et al. (2019), Potapova and Gerton (2019), Warmerdam and Wolthuis (2019)]. Active rDNA repeats are defined as open/accessible chromatin structures, bound by the upstream binding factor (UBF), which is essential for decondensing rDNA chromatin and determining the active rDNA state (Sanij et al., 2008; Hamdane et al., 2014). Active repeats can be either transcriptionally active or transcriptionally competent but non-transcribed, depending on cell cycle phase, cellular signaling, nutrient availability and/or stress stimuli (Schneider, 2012; Xie et al., 2012; Goodfellow and Zomerdijk, 2013; Zhao et al., 2016; Hung et al., 2017). Inhibition of Pol I transcription by loss of the initiation factor RRN3 or upon treatment with the selective inhibitor CX-5461 has no effect on UBF binding nor the proportion of active to inactive rDNA ratio (Quin et al., 2016; Herdman et al., 2017). Thus, UBF binding, not transcription, establishes the active rDNA fraction consistent with (Moss et al., 2019). Inactive rDNA repeats, which lack UBF binding, can be CpG methylated at the rDNA promoter and stably silenced, or non-methylated and hence be in a “pseudo-silent” state (Sanij et al., 2008; Sanij and Hannan, 2009; Hamperl et al., 2013). UBF binding/release is critical for the conversion between active/inactive rDNA repeats, termed rDNA class switching (Sanij et al., 2008; Hamdane et al., 2014; Diesch et al., 2019).

Dysregulated rDNA transcription is linked to a diverse range of human disorders including cancer (Hannan K.M. et al., 2013; Valori et al., 2020). This link is underscored by the modulation of rDNA transcription and rDNA silencing during differentiation and tumorigenesis (Conconi et al., 1989; Sanij et al., 2008; Grummt and Langst, 2013; Hamperl et al., 2013; Hayashi et al., 2014; Savic et al., 2014; Woolnough et al., 2016; Diesch et al., 2019; Prakash et al., 2019). Increased rDNA transcription is a well-known hallmark of cancer. We have shown that a high rDNA transcription rate is required for the oncogene MYC to drive transformation (Chan et al., 2011; Bywater et al., 2012). Thus, rather than being a housekeeping process, rDNA transcription is a key driver of oncogenesis (Hein et al., 2013). Interestingly, variation in the number of rDNA repeats has also been associated with cancer (Stults et al., 2009). This variation in copy number is due to the tandem arrangement of the rDNA rendering it susceptible to recombination, which can result in rDNA instability, such as the loss and gain of rDNA copies. Intriguingly, spontaneous alterations in rDNA organization were over 100-fold elevated in cells lacking Bloom Syndrome protein, a RECQ helicase involved in homologous recombination (HR) DNA repair, and 10-fold elevated in cells lacking ATM (ataxia-telangiectasia, mutated) compared with wild-type controls (Killen et al., 2009). These rDNA alteration phenotypes seem to correlate with the increased cancer predisposition reported in Bloom syndrome and ataxia-telangiectasia patients (Killen et al., 2009). These results suggest that rDNA instability may mediate the predisposition for cancer progression. It has been proposed that reduction of rDNA silencing and rDNA instability underpin global genomic instability, and that this can drive the etiology and progression of cancer (Diesch et al., 2014; Wang and Lemos, 2017; Xu B. et al., 2017; Udugama et al., 2018; Valori et al., 2019). Nevertheless, it appears that increased rRNA production can be achieved even if the rDNA copy number is reduced (Wang and Lemos, 2017), which can occur by regulating the rate of Pol I transcription per rDNA repeat and/or the number of active rDNA repeats.

Pol I transcription is a therapeutic target for small anti-cancer drugs (Bywater et al., 2012; Hein et al., 2013; Peltonen et al., 2014). The first-in-class Pol I transcription inhibitor, CX-5461 is a promising cancer therapy as a single agent and in combination therapy in pre-clinical models of lymphoma, acute myeloid leukemia, prostate and ovarian cancer (Bywater et al., 2012; Devlin et al., 2016; Rebello et al., 2016; Hein et al., 2017; Yan et al., 2019; Sanij et al., 2020). Recently, the sensitivity profile of CX-5461 was shown to closely resemble a topoisomerase II (TOP2) poison (Olivieri et al., 2019; Bruno et al., 2020). TOP2a is an essential component of the Pol I pre-initiation complex (Ray et al., 2013) and while CX-5461 demonstrates highly selective inhibition of Pol I transcription initiation, it is possible that it does so, in part, by trapping TOP2a at the rDNA repeats. Importantly, our first-in-human trial of CX-5461 in patients with advanced hematological cancers established on-target efficacy in targeting Pol I transcription and demonstrated single-agent anti-tumor activity in p53 wildtype and p53-mutant hematologic malignancies (Khot et al., 2019). CX-5461 is also in Phase I clinical trial in solid tumors and has shown preliminary activity in patients with HR deficiency tumors (Xu H. et al., 2017; Hilton et al., 2020). In order to maximize the clinical impact and success of CX-5461, it is crucial to identify biomarkers of response to enable patient stratification.

CX-5461 induces the p53-dependent “nucleolar stress response” (Bywater et al., 2012; Devlin et al., 2016; Pelletier et al., 2018) and a p53-independent checkpoint induced by targeted activation of the DNA-damage response (DDR) upon the induction of chromatin defects and replication stress at the rRNA genes (Quin et al., 2016; Sanij et al., 2020). Activation of each checkpoint results in different cellular phenotypes depending on cell type and cellular context (Yan et al., 2017; Sanij et al., 2020). The nucleolar stress response results in p53-mediated G1/S arrest and apoptosis, with apoptosis being the major response in MYC-driven lymphoma. The p53-independent checkpoint results in S and G2/M phase cell cycle arrest and is the predominant phenotype of solid tumor cells (Quin et al., 2016; Sanij et al., 2020). CX-5461 has been shown to activate ATM/ATR (ataxia telangiectasia and RAD3 related) signaling and a G2/M cell cycle checkpoint in ovarian cancer (OVCA) cells with differential sensitivities observed across a panel of 32 OVCA cell lines (Sanij et al., 2020). OVCA cell lines with higher rates of Pol I transcription are more sensitive to CX-5461 (Sanij et al., 2020). Thus, rDNA copy number and rDNA chromatin status may function as biomarkers of response to inhibition of Pol I transcription.

Here we investigated whether alterations in rDNA copy number and changes in the proportion of active to inactive rDNA repeats correlate with sensitivity to CX-5461 across a panel of OVCA cell lines. We found that an increase in the proportion of active rDNA repeats correlates with increased OVCA cell sensitivity to CX-5461. Further, deleting rDNA copies led to an increase in the proportion of active rDNA repeats, which also correlated with increased sensitivity to CX-5461 and genome-wide instability. Therefore, we propose that an increased fraction of active rDNA repeats is a potential biomarker of response to CX-5461 therapy. Our data also demonstrates that deleting rDNA copies is associated with increased sensitivity to DNA damage highlighting the strong interplay between rDNA and genome-wide instability.

## Materials and Methods

### Cell Culture

Individuality and the identity of OVCA cell lines (listed in Supplementary Tables S1, S2) were confirmed by a PCR-based short tandem repeat (STR) analysis using six STR loci. Cell lines were maintained in culture (Supplementary Table S1) for a maximum of 8–10 weeks. CX-5461 was purchased from Synkinase and 10 mM stocks were prepared in 50 mM NaH_2_PO_4_. Proliferation time course and growth curves for the OVCA cell lines were obtained by assessing cell confluency using the Incucyte ZOOM (Essen Instruments) imaging system. Doubling time for each cell line was calculated using non-linear fit of exponential growth using GraphPad prism software.

### 47S rRNA Expression

For 47S rRNA expression analysis, cells were lysed, RNA extracted, and first-strand cDNA synthesized using random hexamer primers and Superscript III (Invitrogen). Quantitative reverse transcription PCR (qRT-PCR) was performed in triplicate using FAST SYBR Green on the StepOnePlus real-time PCR system (Applied Biosystems, United States). Primer sequences are listed in Supplementary Table S3. Measurement of baseline (basal) rDNA transcription rates of exponentially growing OVCA cell lines was reported in Sanij et al. (2020), determined by qRT-PCR analysis using primers specific to the external transcribed spacer 5’ETS (ETS2). Expression levels in each cell line were normalized to Vimentin mRNA and expressed as fold change relative to TOV112D cells. The mean and standard error of the mean (SEM) values of *n* = 3 biologically independent experiments per cell line were utilized in this study.

### ZFN Gene Editing

Zinc Finger Nucleases (ZFNs) induce double strand DNA breaks (DSBs) at a specific target region, recognized by a zinc finger DNA-binding domain fused with DNA-cleavage domain Gaj et al. (2013). TOV112D cells were transduced with Lentiviruses expressing empty vector or co-transduced with two ZFN targeting rDNA sequences (Supplementary Figure S1) followed by selection with puromycin for 5 days, then FACS to generate clonal cell lines.

### Measurement of rDNA Copy Number

qPCR analysis of 100 ng of genomic DNA (gDNA) was performed in triplicate using FAST SYBR Green on the StepOnePlus real-time PCR system (Applied Biosystems, United States). Primer sequences are listed in Supplementary Table S3. Changes in abundance were to normalized to corresponding Vimentin levels as a single copy locus control and expressed as fold change relative to TOV112D by 2(^–ΔΔC^*^T^*).

For quantification using Southern blotting, gDNA was isolated from 10^6^ cells, digested with *Sal*I, and separated on a 0.9% agarose gel, and alkaline southern blotting was performed. rDNA was visualized using a ^32^P (Amersham)-labeled probe (+1601-2089 base pair relative to transcription start site) within the 5’ETS (external transcribed spacer) region of rDNA and binding detected using a Phosphorimager (GE Healthcare). Signal quantitation was performed using ImageQuant (TLv2005.04; GE Healthcare).

### Psoralen Cross-Linking Assay

Cells were lysed in 10 mM Tris–HCl, pH 7.4, 10 mM NaCl, 3 mM MgCl_2_, and 0.5% NP-40, and nuclei were pelleted, resuspended in 50 mM Tris–HCl, pH 8.3, 40% glycerol, 5 mM MgCl_2_, and 0.1 mM EDTA, and irradiated in the presence of 4,5,8′-trimethylpsoralen (Sigma-Aldrich) with a 366 nm UV light box at a distance of 6 cm (Conconi et al., 1989). 200 μg/ml psoralen was added at 1:20 dilution every 4 min for a total irradiation time of 20 min. gDNA was isolated, digested with *Sal*I, and separated on a 0.9% agarose gel, and alkaline Southern blotting was performed. To reverse psoralen cross-linking, filters were treated with 254 nm UV at 1,875 × 100 μJ/cm^2^ using a UV cross-linker (Stratalinker 2400; Agilent Technologies). The membrane was then hybridized to a purified ^32^P (Amersham)-labeled rDNA probe (+ 1601-2089), visualized by scanning on a PhosphoImager (GE Healthcare), and quantitated using ImageQuant (TLv2005.04; GE Healthcare).

### Chromatin-Immunoprecipitation (ChIP)

Chromatin-immunoprecipitation was performed as described previously (Poortinga et al., 2004; Sanij et al., 2008). Cross-linking was achieved with 0.6% formaldehyde and assays performed using 10^6^ cells per IP. For all ChIPs, 8 μl of sera was used per IP. Samples were analyzed in triplicate using the FAST SYBR green dye on the StepOnePlus real- time PCR system (Applied Biosystems). To calculate the percentage of total DNA bound, unprecipitated input samples from each condition were used as a reference for all qPCR reactions. Primer sequences are listed in Supplementary Table S3.

### Immunofluorescence (IF)

Cells were fixed in 4% paraformaldehyde (10 min at room temperature), permeabilized with 0.3% Triton X-100 in PBS for 10 min on ice, washed with PBS, and blocked with 5% goat serum and 0.3% Triton X-100 in PBS for 30 min. Cells were sequentially incubated with the primary and secondary antibody (Supplementary Table S4), then washed with PBS. Nuclei were counterstained with DAPI in VECTASHIELD mounting media (Vector Labs). Images were acquired on an Olympus BX-61 microscope equipped with a Spot RT camera (model 25.4), using the UPlanAPO 60X, NA 1.2 water immersion objective and Spot Advanced software (v.4.6.4.3). The gamma adjust and background subtract settings for adjusting the image after acquisition were identical for all images.

### rDNA Fluorescent *in situ* Hybridization (FISH)

Following performing IF, slides were fixed in methanol:acetic acid (3:1) for 5 min at room temperature then dehydrated through a 70%–80% ethanol series. Slides were denatured in 70% formamide/2XSSC (saline-sodium citrate) or 10 min at 83°C, then dehydrated through the ethanol series and air-dried. Probes derived from the intergenic spacer of the human ribosomal gene repeat provided by Prof. Brain McStay, NUI Galway. 100 ng of denatured biotin-labeled probe were combined with 30 μg salmon sperm DNA and 18 μg Cot1 carrier DNA (Invitrogen) in 2XSSC with 50% formamide and 20% dextran sulfate and added per slide then hybridized at 37°C for 24 h in a humidified chamber. Slides were washed in 50% formamide/2XSSC at 42°C for 15 min and 0.1XSSC at 60°C for 15 min. Streptavidin-Alexa fluor 488 was added for 1 hr at 37°C and the slides then washed in 0.05% Tween-20/4XSSC for 15 min. Slides were mounted in DAPI. Images were acquired on an Olympus BX-61 microscope as described above.

### COMET Assay

Cells were collected and processed as described in the manufacturer’s protocol (Trevigen, Comet Assay 4250-050-K). Images were acquired on an Olympus BX-61 microscope using the Olympus UPlanAPO 203, NA 1.2 water immersion objective as described above.

### Statistical Analysis

Pearson correlation coefficient, Spearman’s rank correlation coefficient, one-way ANOVA multiple tests and Student’s *t*-test were employed as indicated in figure legends.

## Results

### A Higher Proportion of Active rDNA Repeats Correlates With OVCA Cell Sensitivity to CX-5461

Our aim was to characterize rDNA features (Figure 1B) that correlate with sensitivity to CX-5461 in order to identify possible predictive biomarkers of response to CX-5461. To do this, we employed a panel of established human OVCA cell lines from a range of histological subtypes. We previously reported that the concentration of drug that induces a 50% reduction in cell proliferation (GI50) varied profoundly between individual OVCA cell lines, and these cell lines were defined as resistant or sensitive to CX-5461 if the GI50 was above or below the geometric GI50 median of 360 nM, respectively (Sanij et al., 2020). We also reported that CX-5461 sensitivity correlates with basal rDNA transcription rate (i.e., rDNA transcription rate of exponentially growing cells) (Sanij et al., 2020) (shown here in Figure 1C). Specifically, we demonstrated that sensitive OVCA cell lines exhibited higher rDNA transcription rates than resistant lines. In this study we extend on the earlier work of Sanij et al. (2020) to assess whether differences in basal rDNA transcription rate were associated with rDNA copy number, and therefore whether copy number may explain OVCA cell line sensitivity to CX-5461. To test this, we measured rDNA copy number (dosage) in 15 OVCA cell lines by Southern blotting, and expressed rDNA dosage as fold change relative to the rDNA copy number of TOV112D cells. A wide range of rDNA dosage, from 0.07-fold in OVCAR8 to 3-fold in OVCAR3, were observed (Figure 1D). We also measured rDNA dosage using FISH in 4 OVCA cell lines and showed that the range of rDNA dosage concorded between the two assays confirming the differences in rDNA dosage between the OVCA cell lines (Supplementary Figure S2A). However, despite this wide range in rDNA dosage, we did not observe a correlation between rDNA dosage and CX-5461 GI50 values (Figure 1E). Thus, these results do not support the hypothesis that rDNA copy number directly determines OVCA cell line sensitivity to CX-5461.

**FIGURE 2:**
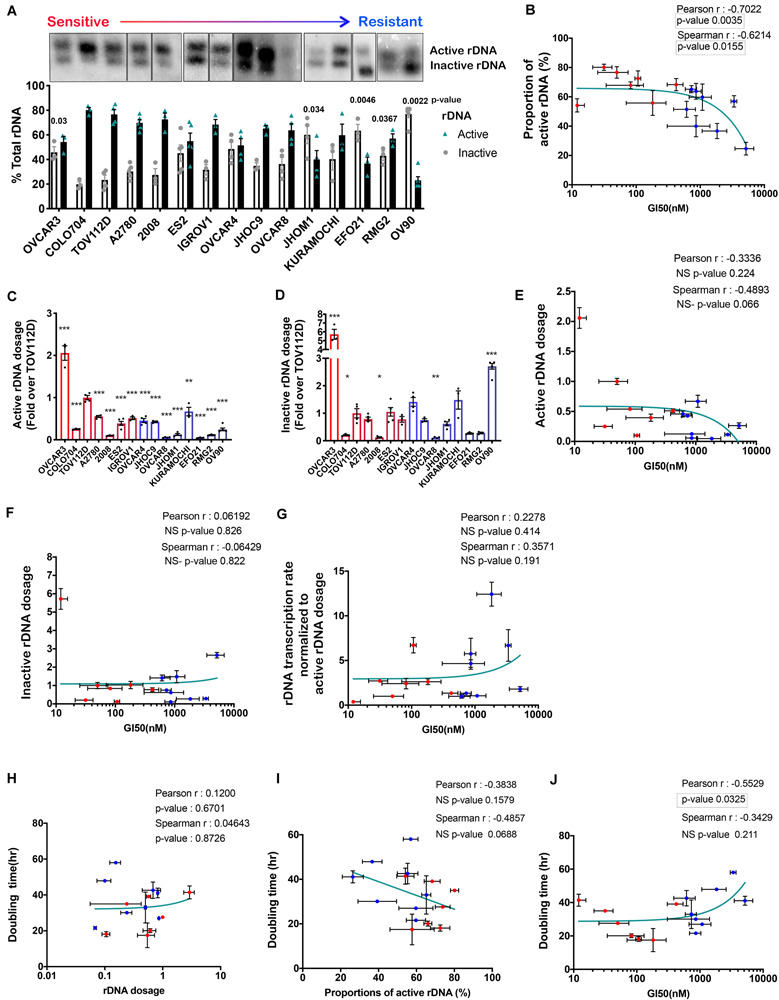
The proportion of active to inactive rDNA chromatin correlates with sensitivity to GI50 by CX-5461 in 15 OVCA cell lines. **(A)** A representative psoralen cross-linking Southern blot analysis of 15 OVCA cell lines. The proportion of active versus inactive rDNA was quantified as a % total rDNA; *n* = 3–4; mean ± SEM. Statistical test of change relative to TOV112D was performed using unpaired *t*-test, *p*-values are indicated. **(B)** Correlation analysis of OVCA cell lines proportion of active rDNA and sensitivity to CX-5461 (GI50). The sensitive cell lines are marked as red dots while the resistant cell lines are blue. Error bars represent mean ± SD. **(C)** The active rDNA dosage and **(D)** inactive rDNA dosage were calculated by multiplying the mean rDNA dosage (Figure 1D) with the mean proportion of active or inactive rDNA, respectively and expressed as fold over TOV112D; *n* = 3, mean ± SEM. Statistical analysis was performed using two-sided one-way ANOVA Dunnett’s multiple comparisons test. *p-value < 0.05, **p-value < 0.01, ***p-value < 0.001, compared to TOV112D. Correlation analysis of OVCA cells sensitivity to CX-5461 (GI50) and: **(E)** active rDNA dosage; **(F)** Inactive rDNA dosage. **(G)** A correlation analysis of OVCA cells sensitivity to CX-5461 (GI50) with rDNA transcription rate normalized to active rDNA dosage [calculated by dividing the basal rate of rDNA transcription in Figure 1C (Sanij et al., 2020) by active rDNA dosage from **(C)**]. **(H)** Correlation analysis of OVCA cell doubling time (Supplementary Table S2) with rDNA dosage, **(I)** the proportions of active rDNA repeats and **(J)** OVCA cells sensitivity to CX-5461 (GI50). Error bars on all correlation graphs represent mean ± SD. Significant *p*-values *p* < 0.05 are highlighted by the rectangles.

We have previously shown that CX-5461 activates nucleolar DDR by inducing chromatin defects and replication stress at the rDNA (Quin et al., 2016; Yan et al., 2019; Sanij et al., 2020). Therefore, we examined whether the number of active rDNA repeats and/or the proportion of active to inactive rDNA repeats are determining factors for CX-5461 sensitivity. To do this, we performed psoralen cross-linking followed by Southern blotting of gDNA from the 15 OVCA cell lines. Actively transcribed and open chromatin rDNA states are accessible to psoralen crosslinking, thus active repeats migrate slower than inactive repeats during agarose gel electrophoresis, allowing the proportion of active/inactive repeats to be quantified (Conconi et al., 1989; Sanij et al., 2008). The relative proportion of active to inactive rDNA repeats varied substantially across the OVCA cell lines (Figure 2A and Supplementary Figure S2B). We found that CX-5461-sensitive cell lines exhibited higher relative proportions of active rDNA repeats compared to resistant cell lines (Figure 2B). We considered whether this sensitivity simply reflects the absolute number of active rDNA repeats. To test this, we calculated active rDNA dosage by normalizing the proportion of active repeats to the relative rDNA dosage in Figure 1D. Interestingly, OVCA cell line sensitivity to CX-5461 showed a trend in correlation with the active rDNA dosage (Figures 2C,E) but not the inactive rDNA dosage (Figures 2D,F). Since sensitivity to CX-5461 correlated with higher basal rate of rDNA transcription (Figure 1C; Sanij et al., 2020), we examined whether this also correlated with parameters of rDNA chromatin activity (Supplementary Figures S3A–C). Basal rate of rDNA transcription did not correlate with rDNA dosage nor the proportion of active to inactive repeats (Supplementary Figures S3A,B) but did show a trend in correlation with active rDNA dosage (Supplementary Figure S3C). Therefore, we tested whether OVCA cell line sensitivity to CX-5461 correlates with rDNA transcription rate normalized to active rDNA dosage, but found no correlation with sensitivity to CX-5461 (Figure 2G). Thus, of all the rDNA activity parameters examined, only the ratio of active to inactive genes and the basal rate of rDNA transcription (Sanij et al., 2020) correlated with CX-5461 sensitivity in OVCA cell lines. However, we cannot dismiss that the technically challenging nature of the assays examined lead to variabilities that may limit our ability to detect correlations.

Recent studies proposed that variation in rDNA copy number is an adaptive response to DNA replication stress, specifically allowing cells with reduced rDNA copy number to rapidly complete replication and cell cycle progression (reviewed in Salim and Gerton, 2019). Therefore, we investigated whether rDNA dosage or the proportion of active to inactive rDNA repeats correlated with OVCA cell line doubling time (Supplementary Table S2). While we observed no correlation between rDNA dosage and OVCA cell line doubling time (Figure 2H), a trend in correlation between doubling time and the proportion of active rDNA repeats (Figure 2I) and sensitivity to CX-5461 (Figure 2J) was observed. Our data therefore suggests that OVCA cell lines with a higher proportion of active rDNA repeats proliferate faster (Figure 2I), and are more sensitive to CX-5461 (Figure 2J). As replication of active rDNA chromatin occurs in early S phase whereas the silent repeats are replicated from mid to late S-phase (Li et al., 2005), it is plausible that cells with elevated proportions of inactive rDNA require a longer S phase in order to complete DNA replication and thus exhibit a longer doubling time.

**FIGURE 3:**
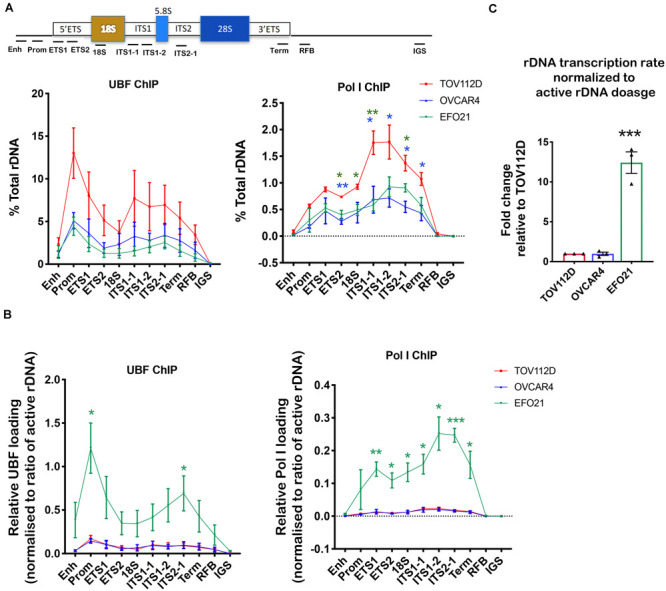
Determining UBF and Pol I occupancy on the rDNA in OVCA cell lines with differential rDNA dosage. **(A)** Quantitative ChIP analysis of UBF and Pol I (POLR1A subunit) loading across the rDNA repeat. The % of total rDNA immunoprecipitated (IP) with the UBF or POLR1A antibodies relative to input control after subtracting background (DNA IP with rabbit sera); Error bars represents mean ± SEM, *n* = 3. Statistical analysis was performed using two-sided unpaired *t*-test. *p-value < 0.05, **p-value < 0.01, OVCAR4 (blue) and EFO21 (green) compared to corresponding TOV112D (red) values. **(B)** UBF and Pol I loading were normalized to the mean proportion of active rDNA as determined by psoralen cross-linking in Figure 2A. Error bars represent mean of *n* = 3 ± SEM. Statistical analysis was performed using two-sided unpaired *t*-test. *p-value < 0.05, EFO21 compared to corresponding TOV112D values. **(C)** The basal rDNA transcription rate normalized to active rDNA dosage was calculated by multiplying the basal rate of rDNA transcription from Figure 1C (Sanij et al., 2020) with the mean values of active rDNA dosage (Figure 2C) and presented as a relative fold change to that for TOV112D. Error bars represent mean ± SEM, *n* = 3, statistical analysis was performed using two-sided unpaired *t*-test,***p-value < 0.001 compared to TOV112D.

UBF has an essential role in establishing and maintaining active rDNA chromatin (Sanij et al., 2008, 2015). To test whether UBF loading on rDNA correlates with sensitivity to CX-5461, we examined UBF and Pol I loading at the rDNA in TOV112D, OVCAR4 and EFO21 cell lines. These cell lines were chosen as they exhibit differential sensitivity to CX-5461 and also vary with respect to rDNA dosage and the ratio of active to inactive rDNA repeats (Figures 1D, 2A). The TOV112D cell line, which has higher rDNA dosage and proportion and of active rDNA than the other two cell lines, also showed a higher occupancy of UBF and Pol I across the transcribed region of the rDNA, while UBF and Pol I rDNA occupancy did not differ between EFO21 and OVCAR4 (Figure 3A). Since UBF and Pol I bind to the active, psoralen-accessible rDNA repeats, their occupancy was normalized to the proportions of active rDNA (Figure 3B). EFO21, which was the least sensitive to CX-5461 (Sanij et al., 2020) and had the lowest active rDNA dosage (Figure 2C) of the three OVCA cell lines, had the highest loading of UBF and Pol I normalized to the proportions of active rDNA (Figure 3B). Thus, while UBF binding defines active repeats, the amount of UBF binding per repeat can vary. Consistent with the elevated UBF and Pol I loading, this cell line also has a higher rate of Pol I transcription normalized to active rDNA dosage compared to the other cell lines tested (Figure 3C). Therefore, the data suggests that the level of Pol I loading and transcription rate normalized to the proportions of active rDNA do not influence sensitivity to CX-5461, but rather sensitivity to CX-5461 is determined by the proportion of active to inactive rDNA repeats and the basal rate of rDNA transcription (i.e., total rDNA transcription output).

### Reducing rDNA Copy Number Increases the Proportion of Active rDNA Repeats and Is Associated With Elevated Genomic Instability

**FIGURE 4:**
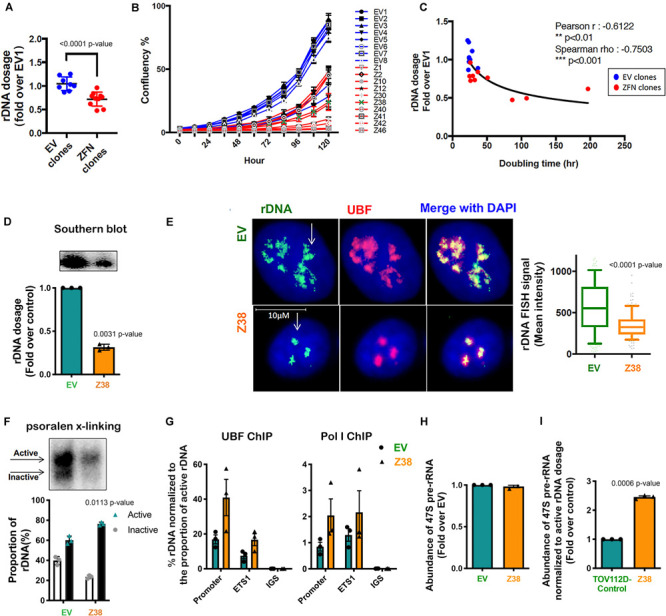
Reducing rDNA copy number is associated with an increased proportion of active to inactive rDNA chromatin and rate of rDNA transcription in the remaining rDNA pool. **(A)** TOV112D cells were infected with Lentivirus expressing empty vector (EV) or 2 ZFNs targeting rDNA sequencing and clonal cell lines generated by puromycin selection. gDNA was extracted from 8 EV and 10 ZFNs exponentially growing clones and rDNA dosage measured by qPCR using the 5’ETS (ETS2) primers, then normalized to Vimentin as a single copy locus control (Supplementary Table S3). Data is represented as fold change over EV1; *** indicates *p* < 0.001 according to unpaired *t*-test. **(B)** The proliferation rate of EV and ZFN clonal cell lines was monitored and the % of cell confluency determined using the IncuCyte; *n* = 3 of technical replicates, mean ± SD. **(C)** Doubling time of cell lines was determined using the IncuCyte measurements and analyzed using GraphPad prism. Correlation analysis of rDNA dosage **(A)** and doubling time for the EV and ZFN clones was performed; Pearson’s r is -0.61, ** indicates *p* < 0.01; Spearman’s rho is -0.75, ***p < 0.001. **(D)** A representative rDNA Southern blot from EV and Z38 cells (upper panel) with quantitation expressed as fold over control (EV); mean ± SEM of *n* = 3 (lower panel). Paired *t* test analysis was performed. **(E)** IF-FISH analysis of rDNA (green: white arrows) and UBF (pink) and DAPI (blue) stained EV and Z38 cells. The intensity of rDNA FISH signal was quantitated using Definiens Tissue Software (Definiens) and graphed as mean ± SD of *n* = 150 cells analyzed over 3 biological replicates, *** indicates *p* < 0.001 according to two-sided Mann-Whitney *t*-test. **(F)** A representative of psoralen cross-linking (x-linking) analysis of EV and Z38 cells (upper panel) and quantitation of *n* = 3; mean ± SEM (lower panel). Paired *t* test analysis was performed. **(G)** qChIP analysis of UBF and Pol I (POLR1A subunit) loading on the rDNA. UBF and Pol I enrichment was calculated as described in Figure 3A and normalized to the mean proportion of active rDNA as determined by psoralen cross-linking in **(F)**, mean ± SEM of *n* = 3. **(H)** The abundance of the 47S pre-rRNA was measured by qRT-PCR and expressed as fold change over control (EV); mean ± SEM of *n* = 3. Paired *t* test analysis was performed. **(I)** The basal rate of rDNA transcription normalized to active rDNA dosage in EV and Z38 cells was calculated by multiplying the rate of rDNA transcription in **(H)** with the mean active rDNA dosage from **(D,F)** and expressed as fold over control (EV); mean ± SEM of *n* = 3. Paired *t* test analysis was performed.

To obtain independent evidence supporting a role for the proportion of active to inactive rDNA repeats in mediating sensitivity to CX-5461, we reduced the rDNA copy number, which has been shown in yeast to mediate an increase in the activity of the remaining repeats (French et al., 2003; Ide et al., 2010). We did this by utilizing zinc-finger nuclease (ZFN) genome-editing. Two pairs of ZFNs were designed to specifically induce double strand DNA breaks (DSBs) targeting the intergenic spacer (IGS) region upstream and downstream of the transcribed unit of the rDNA repeat thus mediating a loss in rDNA copy number (Supplementary Figure S1). Copy number was measured by qPCR in eight empty vector (EV) transduced TOV112D cell lines and ten ZFN clonal TOV112D cell lines, then expressed as fold change relative to the first EV cell line (EV1). A robust decrease in rDNA copy number (∼20–50%) was observed in the ZFN expressing cell lines compared to EV controls (Figure 4A). We measured the effect of reducing the rDNA copy number on cell proliferation using the IncuCyte (Figures 4B,C). Overall, the ZFN cell lines exhibited reduced proliferation and longer doubling times (25.6 to 196.8 h) compared to the EV clones (21.9 to 34.9 h), suggesting that the ZFN-mediated reduction in rDNA copy number leads to defects in proliferation. However, it is also plausible that the ZFNs induced multi-DSBs within the rDNA loci leading to activation of a DNA damage response and growth inhibition.

We next evaluated a ZFN clone (Z38) that exhibited a ∼68% reduction in rDNA copy number compared to the EV cell line (Figure 4D). Quantitation of rDNA copy number performed using Southern blotting (Figure 4D) and rDNA-FISH combined with IF for UBF (Figure 4E) confirmed the reduced rDNA dosage in Z38 cells compared to EV cells. Psoralen cross-linking assays demonstrated a higher proportion of active rDNA in Z38 cells compared to EV cells (76% compared to 60%; Figure 4F), which was associated with an increase in UBF and Pol I occupancy normalized to the proportion of active rDNA (Figure 4G). However, the EV and Z38 cells displayed similar rates of basal rDNA transcription (Figure 4H). Together, the data suggest that as a consequence of reducing rDNA copy number, the proportion of active rDNA repeats increases, concomitant with an increase in Pol I transcription rate normalized to active rDNA dosage (Figure 4I), to maintain total rDNA transcriptional output (Figure 4H).

**FIGURE 5:**
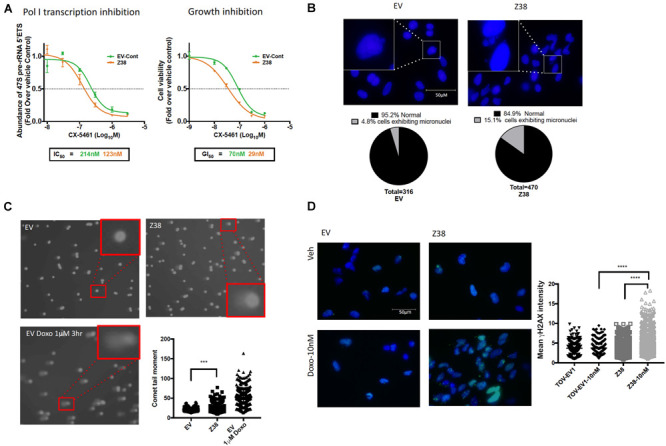
Z38 cells with reduced rDNA copy number exhibit a higher sensitivity to CX-5461 and doxorubicin compared to EV control cells. **(A)** analysis of Pol I transcription inhibition by CX-5461 in EV control (EV-Cont) and Z38 cell lines (left panel). Cells were treated with increasing concentrations of CX-5461 for 1 h and the abundance of 47S pre-rRNA determined by qRT-PCR. IC50 of Pol I transcription inhibition for each cell lines was determined using GraphPad prism; *n* = 3; mean ± SEM. Analysis of growth inhibition by CX-5461 in EV and Z38 cell lines (right panel). Cells were treated with increasing concentrations of CX-5461 for 48 h and the cell viability (PI staining) was measured using IncuCyte; *n* = 3; mean ± SEM. **(B)** Z38 cells exhibit higher basal level of micronuclei formation. Representative images and quantitation of % of cells with micronuclei, *n* = 1. **(C)** Representative images of alkaline comet assay in EV and Z38 cell lines for detecting basal DNA damage levels. EV cells were treated with 1 μM Doxorubicin for 3 h as a positive control for DNA damage. Quantitation of comet tail moment was performed using OpenComet v.1.3 software; *n* = 3, mean ± SEM, statistical significance determined using one-way ANOVA, ** indicates *p* < 0.01. **(D)** IF analysis of γH2AX foci as a marker of DSBs in EV and Z38 cells treated with vehicle (Veh) or Doxorubicin (Doxo-10nM) for 3 h. Quantitation of the mean signal intensity was determined using Definiens of *n* = 245 cells analyzed over two biologically independent experiments, mean ± SD. Statistical analysis was performed using one-way ANOVA multiple comparisons, **** indicates *p* < 0.0001.

We next determined the sensitivity of Z38 cells to CX-5461. Exponentially growing cells were treated with increasing concentrations of CX-5461 for one hour followed by determination of 47S rRNA abundance. Interestingly, CX-5461 IC50 values for Pol I transcription inhibition by CX-5461 decreased by 50% in the Z38 clone (123 nM) compared to EV (214 nM) (Figure 5A). Thus, Z38 cells are more sensitive to Pol I transcription inhibition by CX-5461 than the control cells (EV). We also determined the rate of proliferation 48 h after treatment. Similarly, GI50 for CX-5461 in Z38 cells (29 nM) was reduced by 60% compared to EV cells (70 nM) (Figure 5A). Thus, the increase in the proportion of active rDNA repeats in Z38 cells is associated with increased sensitivity to CX-5461. This is consistent with the correlation between the ratio of active to inactive rDNA repeats and sensitivity to CX-5461 (Figures 2A,B).

Several studies suggest a strong correlation between rDNA chromatin activity status and genome integrity (reviewed in Diesch et al., 2014). In mouse cells, loss of rDNA silencing results in destabilization of the perinucleolar heterochromatin, which is crucial for ensuring genome stability (Guetg et al., 2010). In addition, yeast with low-copy strains have impaired DNA damage repair during S-phase and consequently higher sensitivity to DNA damaging agents such as ultraviolet radiation and methyl methanesulfonate (Ide et al., 2010). Hence, variation in rDNA chromatin states may predispose cells to genomic instability and influence cellular responses to DNA damage. To investigate this possibility, we assessed micronuclei formation, a marker of genome instability, and used the alkaline comet assay to measure single strand DNA breaks, DSBs and other DNA lesions at a single cell level in Z38 cells. The Z38 cells exhibited a higher abundance of micronuclei formation (Figure 5B) and significantly brighter and longer comet tails (Figure 5C) compared to the EV cells, suggesting a higher degree of basal DNA damage although at a lower level compared to EV cells treated with doxorubicin. Furthermore, while the basal level of phosphorylated histone variant (γH2AX), a marker of DNA damage, was similar in the Z38 and EV cells, low dose doxorubicin (Doxo, 10 nM) treatment for 3 h mediated a significant increase in γH2AX foci staining specifically in the Z38 cells (Figure 5D). Thus, Z38 cells with a higher proportion of active rDNA repeats exhibit greater sensitivity to DNA damage mediated by doxorubicin. Altogether, these results suggest that a reduction in rDNA copy number by ZFNs mediates an increase in the ratio of active to inactive rDNA repeats and enhances the cells sensitivity to CX-5461 and DNA damage.

## Discussion

In this report, we utilized a panel of human OVCA cell lines to identify potential predictive biomarker(s) of therapeutic response to CX-5461. Our data revealed that sensitivity to CX-5461 significantly correlates with the basal rDNA transcription rate (total rDNA transcriptional output), the proportion of active to inactive rDNA repeats and doubling time. Our analyses also showed a correlation trend for sensitivity to CX-5461 with active rDNA dosage, but there was no correlation between CX-5461 sensitivity and rDNA dosage (copy number), inactive rDNA dosage or rDNA transcription rate normalized to active rDNA dosage. However, we cannot exclude that the high variation in rDNA copy number and proportion of active rDNA repeats measurements may limit our ability to detected significant correlations. Such correlations may be revealed in time should methods that more precisely measure these parameters be developed.

The strong association of higher proportions of active rDNA with sensitivity to growth inhibition by CX-5461 is consistent with CX-5461’s mode of action in triggering defects associated with open chromatin and replication stress at the rDNA (Quin et al., 2016; Yan et al., 2019; Sanij et al., 2020), including potentially acting as a TOP 2 poison (Bruno et al., 2020) selectively at the rDNA and/or across the genome. We have demonstrated that CX-5461 activates nucleolar ATM and ATR leading to activation of cell cycle checkpoints and global replication-mediated DNA damage (Quin et al., 2016; Yan et al., 2019; Sanij et al., 2020). Our data therefore suggests that cells with a higher ratio of active rDNA are more sensitive to CX-5461-mediated nucleolar DDR and activation of cell cycle checkpoints, with faster proliferating cells being more responsive to cell cycle arrest.

We found that the proportion of active rDNA repeats does correlate with OVCA cell doubling time. This finding is important in the context of recent bioinformatic analyses of whole genome sequencing data demonstrating that rDNA repeats tend to be lost in cancers (Wang and Lemos, 2017; Xu H. et al., 2017). These results are consistent if we assume that the lower rDNA copy number reported for these cancers has resulted in an increased proportion of active rDNA repeats. However, we also found that rDNA copy number did not correlate with doubling time (Figure 2H), suggesting that the relationship between copy number and proliferation time is more complex, which might reflect the multiple ways cells can achieve a certain level of rDNA transcription. Consistent with this, we also showed that the baseline rate of rDNA transcription does not correlate with the proportion of active rDNA repeats or rDNA dosage, although there is a trend in correlation with active rDNA dosage (Supplementary Figure S3), possibly due to the rDNA transcription rate being dependent on both the number of active rDNA repeats and the density of Pol I loading/transcription elongation at these active repeats (Conconi et al., 1989; French et al., 2003; Schneider, 2012; Hung et al., 2017). In this case, the increased rate of rDNA transcription observed in cancer cells (Drygin et al., 2010; Hannan R.D. et al., 2013) could be mediated independently of rDNA copy number. We therefore propose that an increase in the proportion of active rDNA repeats is likely to be a more consistent phenotype of proliferative cancers than a reduction in rDNA copy number.

We demonstrated that rDNA copy number can be reduced using dual ZNF targeting. Whether this occurred via precise deletion of whole rDNA repeats, thus by homologous recombination-mediated repair of DSBs, or by non-homologous end joining of the break sites remains unclear. Distinguishing between these two possibilities requires sequencing of the ZNF clones, but the interpretation is likely to be complicated by reports suggesting there are pre-existing incomplete units in the human rDNA (Caburet et al., 2005). The reduced rDNA copy number in the ZFN-targeted TOV112D cells is associated with an increased proportion of active rDNA repeats, in agreement with previous reports in yeast showing altering rDNA copy number modulated rDNA chromatin states (Ide et al., 2010). The increased proportion of active rDNA repeats may result from rDNA class switching, specifically the reduced pool of rDNA repeats promotes UBF binding to any inactive repeats thus converting them to active repeats (Diesch et al., 2019). Alternatively, ZNF deletion may selectively target inactive rDNA repeats. However, CRISPR Cas9-mediated DSBs preferentially occurs in euchromatic regions in the genome, suggesting gene editing is more efficient in euchromatin than in heterochromatin (Jensen et al., 2017). Furthermore, dynamic switching between active and inactive repeats was reported upon ATRX depletion, which resulted in reduced rDNA silencing, specific loss of the inactive rDNA repeats and increased proportions of active rDNA repeats (Udugama et al., 2018) thus making the distinction in genome editing bias toward active or inactive rDNA repeats largely void. Importantly, our data demonstrates that the reduction in rDNA copy number observed in Z38 cells induced genome-wide instability, as illustrated by increased micronuclei formation and other markers of DNA damage (Figure 5). Furthermore, Z38 was more sensitive to doxorubicin-induced DNA damage, consistent with results in yeast showing reduced rDNA copy number increases sensitivity to DNA damaging agents (Ide et al., 2010). Whether this increased genomic instability is mediated by the reduced rDNA copy number, the increased proportion of active rDNA repeats, or another feature due to ZNF treatment remains to be determined.

Taken together, this study demonstrates a significant correlation between OVCA sensitivity to CX-5461 and the proportion of active to inactive rDNA repeats. These data suggest that rDNA chromatin states may be a useful biomarker for sensitivity to targeted Pol I transcription therapies. Validation of this parameter as a predictive biomarker of response to CX-5461 in patient samples in future clinical trials will be important to translate these findings to the clinic. A potential barrier to the effectiveness of rDNA chromatin status as a biomarker is the lack of precision with which the proportion of active rDNA repeats can currently be determined, however our results suggest there is value in developing improved methods for measuring rDNA activity state *in situ.*

## Author Contributions

ES, RH, and AG conceptualized and designed the study. ES, KH, GP, NH, DC, and KS developed the methodology. JS and ES acquired the data. JS, ES, KH, RH, AG, and RP analyzed and interpreted the data. ES, RH, RP, and AG supervised the study. All authors wrote, reviewed, and/or revised the manuscript.

## Conflict of Interest

RH is a Chief Scientific Advisor to Pimera Inc.

The remaining authors declare that the research was conducted in the absence of any commercial or financial relationships that could be construed as a potential conflict of interest.
