# Supplementary material for: rDNA Chromatin Activity Status as a Biomarker of Sensitivity to the RNA Polymerase I Transcription Inhibitor CX-5461
Source: Front Cell Dev Biol. 2020 Jul 3;8:568. doi: 10.3389/fcell.2020.00568 (PMC7349920; doi:10.3389/fcell.2020.00568)
Supplement: Supplementary file 1 [file Data_Sheet_1.PDF]

Supplementary Figure 1

A

Region	Position base	Zinc Finger Nuclease Binding/cutting site
Upstream target	-999 to -955	5'CGGGAAGAGCTTCTCGACTCacggtTTCGCTTTCGCGGTCCACG3'
Downstream target	+14,347 to +14,387	5'AGCCGGACCCGCCGCgtccccGTCTCGGTCGGCACCTCCG3'

B

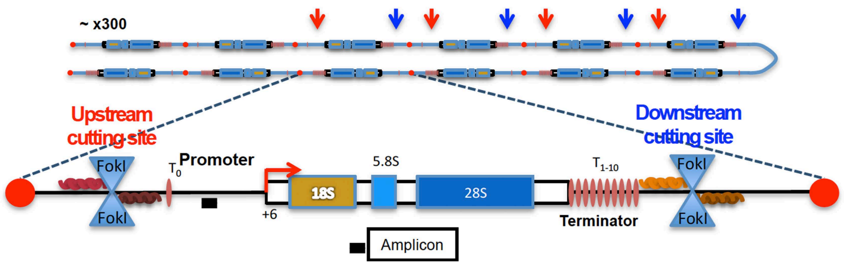

C

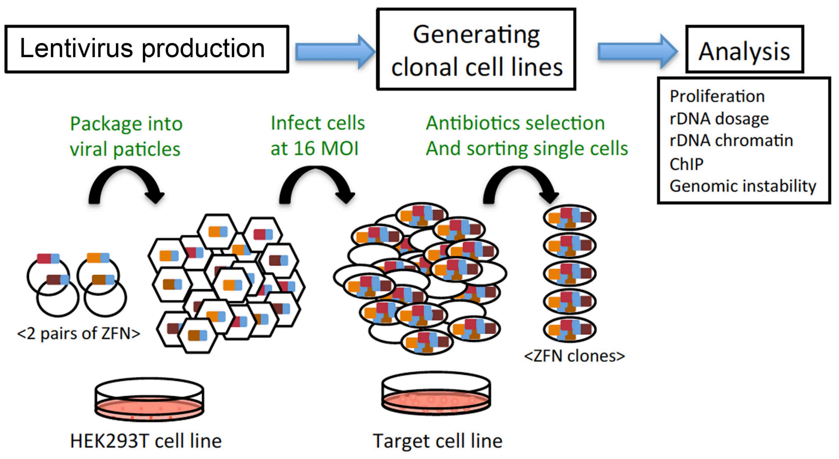

Supplementary Figure 1. Targeting the rDNA loci using ZFN to reduce rDNA copy number. **(A)** rDNA sequences targeted by ZFNs. ZFNs consist of a Zinc-Finger DNA binding motif and Fok-I endonucleases that target the rDNA sequences. **(B)** A schematic of the human rRNA gene cluster detailing the ZFN target sites within the IGS, upstream and downstream of the rDNA transcribed region. Arrows indicate the ZFNs target site; red arrows for upstream site of the transcribed region and blue for the downstream site. **(C)** A schematic of the experimental protocol. Lentivirus expressing 2 pairs of the ZFNs (ZFN) or empty vector (EV) were transduced into HEK293T cells. The concentration of lentivirus was determined using the qRT-PCR based Lenti-X qRT-PCR Titration Kit (Takara Cat# 631235) according to manufacturer's instructions used to infect the TOV112D cell line with a MOI (multiplicity of infection) of 16. EV and ZFN cells were selected with 1μg/ml puromycin for 5 days, followed by single cell sorting using flow cytometry to generate clonal cell lines.

Supplementary Figure 2

A

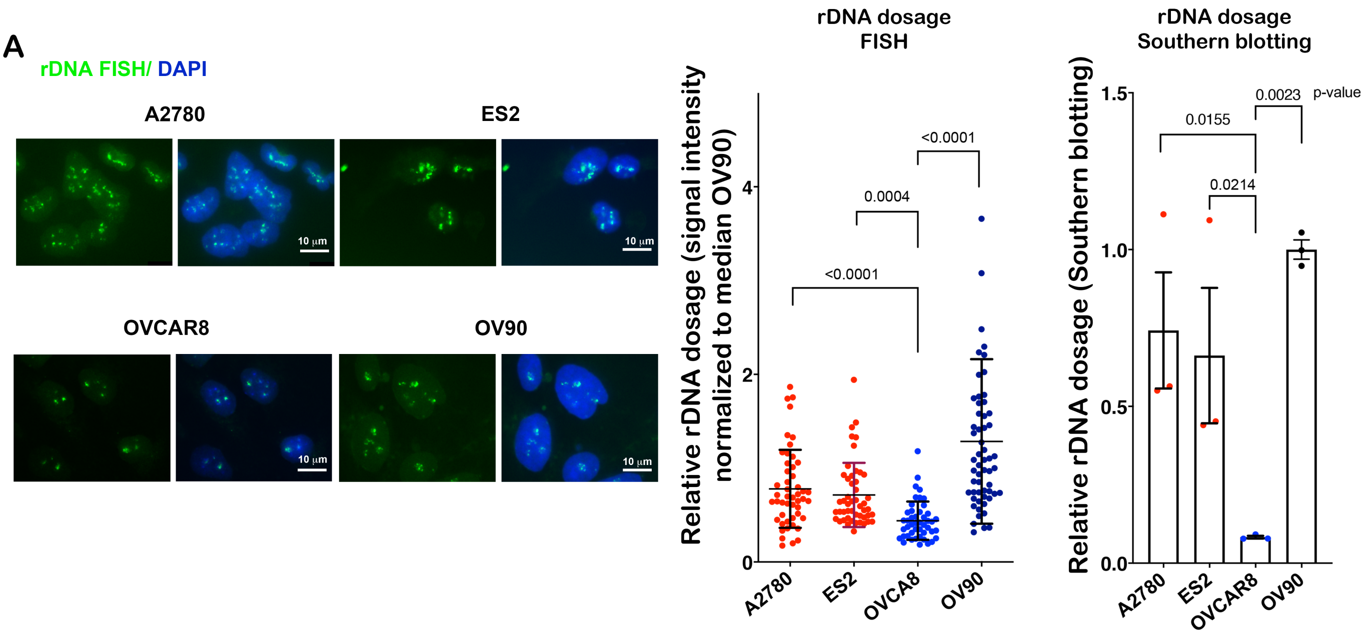

B

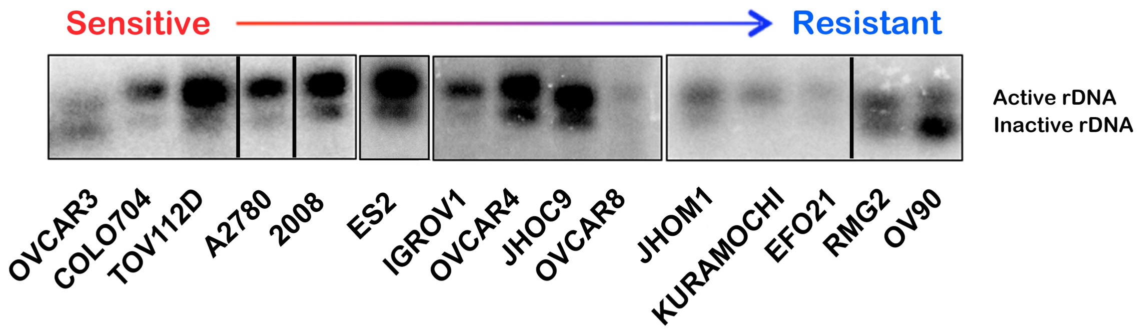

Supplementary Figure 2. (A) rDNA FISH analysis of 4 exponentially growing OVCA cell lines. The intensity of rDNA FISH signal was quantitated using Definiens Tissue Software (Definiens) and graphed as mean +/- SD of 50 cells analysed over two biologically independent experiments. Statistical analysis was performed using two-sided one-way ANOVA Kruskal-Wallis multiple comparison test (left graph). The graph on the right represents the rDNA dosage, as in Figure 1D, expressed as fold relative to median OV90; n=3; mean +/- SEM. Statistical analysis was performed using two-sided one-way ANOVA multiple comparisons test. **(B)** A representative analysis of **n=3-4** psoralen cross-linking Southern blot analysis of 15 OVCA cell lines as in Figure 2A.

Supplementary Figure 3

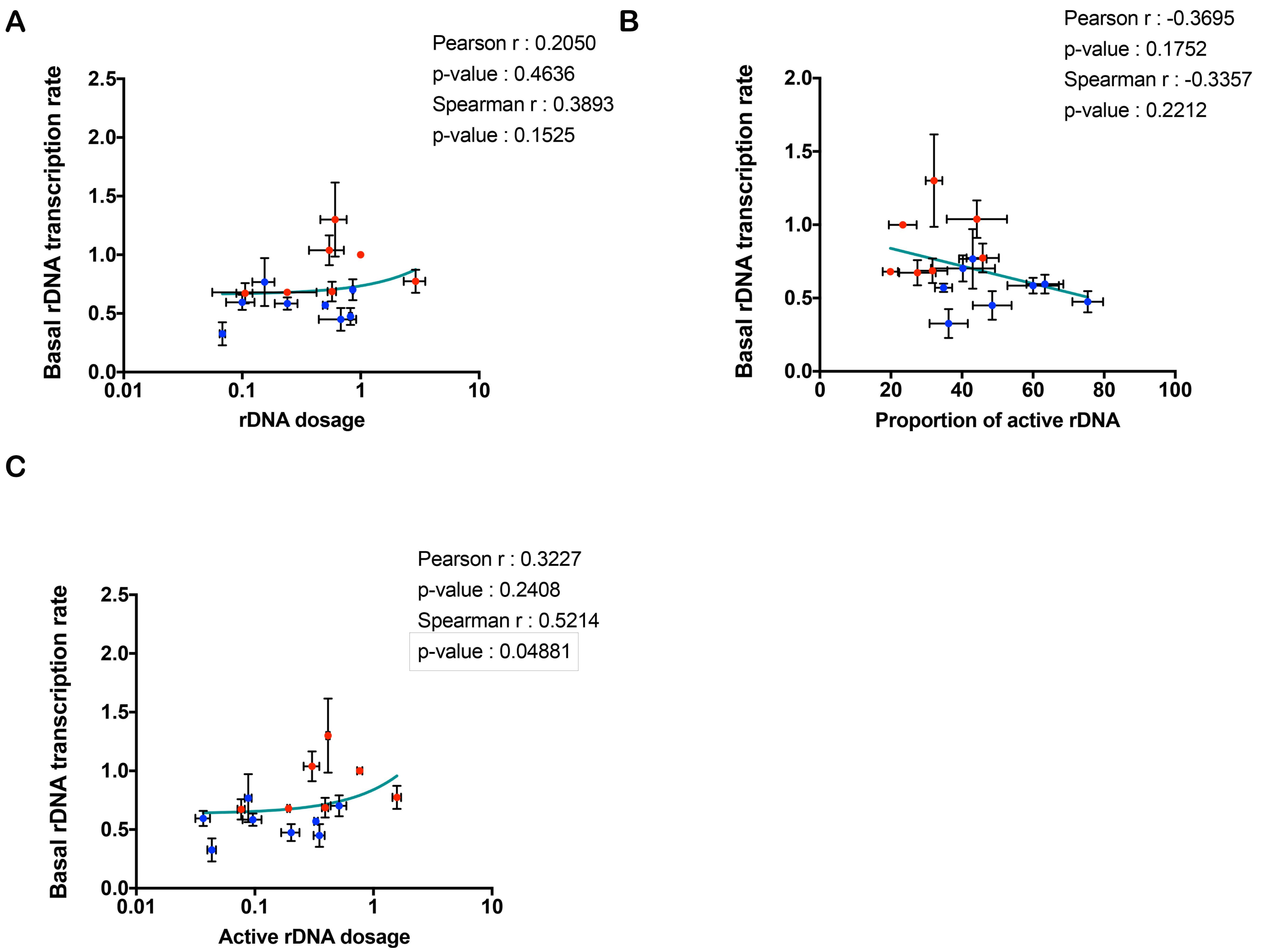

Supplementary Figure 3. Correlation analysis of the basal rDNA transcription rates of OVCA cell lines (Figure 1C) and reported in (Sanij et al., 2020) with **(A)** rDNA dosage; **(B)** the proportion of active rDNA repeats; and **(C)** Active rDNA dosage. The CX-5461-sensitive cell lines are marked as red dots while the resistant cell lines are in blue. Error bars represent mean +/- SD. Significant p-values $p<0.05$ is highlighted a fine rectangle.

Supplementary Table 1. OVCA cell lines sources and culture conditions

<table>
<tr><th>Cell Line</th><th>Histology</th><th>Source</th><th>Culture Conditions</th></tr>
<tr><td>2008</td><td>Endometrioid</td><td>Stephen Howell at University of California, San Diego</td><td rowspan="9">RPMI-1640 plus HEPES supplemented with 10% (v/v) FBS, glutamax and 1% (v/v) antibiotics / antimycotics</td></tr>
<tr><td>A2780</td><td>Adenocarcinoma Serous</td><td>European Collection of Cell Cultures</td></tr>
<tr><td>COLO704</td><td>Adenocarcinoma</td><td>Deutsche Sammlung von Mikroorganismen und Zellkuturen</td></tr>
<tr><td>ES2</td><td>Serous</td><td>American Type Culture Collection</td></tr>
<tr><td>IGROV1</td><td>Endometriod, serous, clear cell</td><td>National Cancer Institute</td></tr>
<tr><td>OVCAR3</td><td>Serous</td><td>National Cancer Institute</td></tr>
<tr><td>OVCAR4</td><td>Serous</td><td>National Cancer Institute</td></tr>
<tr><td>OVCAR8</td><td>Serous</td><td>National Cancer Institute</td></tr>
<tr><td>KURAMOCHI</td><td>Serous</td><td>Health Science Research Resources Bank</td></tr>
<tr><td>OV90</td><td>Serous</td><td>American Type Culture Collection</td><td rowspan="2">MCDB 105 : M199 plus HEPES, 10% (v/v) FBS, 1% (v/v) antibiotics / antimycotics</td></tr>
<tr><td>TOV112D</td><td>Endometrioid</td><td>American Type Culture Collection</td></tr>
<tr><td>JHOC9</td><td>Clear Cell</td><td>RIKEN</td><td rowspan="2">DMEM : F12 plus HEPES, 10% (v/v) FBS, MEM non-essential amino acids, 1% (v/v) antibiotics/ antimycotics</td></tr>
<tr><td>JHOM1</td><td>Mucinous</td><td>RIKEN</td></tr>
<tr><td>RMGII</td><td>Clear Cell</td><td>Health Science Research Resources Bank</td><td>F12, 10% (v/v) FBS, 1% (v/v) antibiotics / antimycotics</td></tr>
<tr><td>EFO21</td><td>Serous</td><td>Deutsche Sammlung von Mikroorganismen und Zellkuturen</td><td>RPMI-1640 plus HEPES, 20% (v/v) FBS, sodium pyruvate, 1% (v/v) antibiotics / antimycotics</td></tr>
</table>

Supplementary Table 2. OVCA cell lines doubling time.

Cell Line	Doubling time (hours)	SD	N
**2008**	18.1	1.4	4
**A2780**	20.1	1.1	3
**COLO704**	35		1
**EFO21**	47.9		1
**ES2**	17.5	6.9	2
**IGROV1**	39.1		1
**JHOC9**	33	8.6	5
**JHOM1**	30.1		1
**KURAMOCHI**	27		1
**OV90**	41.1	2.7	3
**OVCAR3**	41.4	3.6	3
**OVCAR4**	42.6	4.6	2
**OVCAR8**	21.6		1
**RMGII**	58		1
**TOV112D**	27.6		1

Supplementary Table 3: Primer sequences for q-RT-PCR, qPCR and PCR.

Application	Target/ location with rDNA relative to transcription start site	Direction	Sequence
qRT-PCR	VIMENTIN	F	AGA GAA CTT TGC CGT TGA AGC T
		R	GAA GGT GAC GAG CCA TTT CC
PCR	ZFN-1	F	CCG TGG GTT GTC TTC TGA CT
		R	CTT TCC GGA GCT CTG CCT A
PCR	ZFN-2	F	GCG CCT TCT CGG CTC TTG AG
		R	CGC GTG AAC GGG AAT CAG CG
ChIP qPCR	Enhancer (Enh) 41982 - 42075 bp	F	AGA GGG GCT GCG TTT TCG GCC
		R	CGA GAC AGA TCC GGC TGG CAG
ChIP qPCR	Promoter (Prom) 42943- 43 bp	F	CCC GGG GGA GGT ATA TCT TT
		R	CCA ACC TCT CCG ACG ACA
ChIP qPCR	ETS1 413 – 521 bp	F	GCT CTT CGA TCG ATG TGG TGA CG
		R	CGG GCG GAG CGA GAA GGA C
qRT-PCR & ChIP qPCR	ETS2 952 -1030 bp	F	GGC GGT TTG AGT GAG ACG AGA
		R	ACG TGC GCT CAC CGA GAG CAG
ChIP qPCR	18S 3990 – 4092 bp	F	CGA CCC ATT CGA ACG TCT
		R	CTC TCC GGA ATC GAA CCC TGA
ChIP qRT-PCR	ITS1-1 5693 – 5773 bp	F	GAG AAC TCG GGA GGG AGA C
		R	GAC ACG CCC TTC TTT CTC TC
ChIP qPCR	ITS2-1 6995 -7104 bp	F	GAG AGA GAC GGG GAG GGC GG
		R	CCG AGG GAG GAA CCC GGA CC
ChIP qPCR	28S 8204 – 8300 bp	F	AGT CGG GTT GCT TGG GAA TGC
		R	CCC TTA CGG TAC TTG TTG ACT
ChIP qPCR	Terminator (Term) 12855 -12970 bp	F	ACC TGG CGC TAA ACC ATT CGT
		R	GGA CAA ACC CTT GTG TCG AGG
ChIP qPCR	Replication fork barrier (RFB) 13534-13790 bp	F	GTGTAGGAGTGCCCGTCG
		R	AAATGTGGGAGAGGGAGTTC
ChIP qPCR	IGS 18155 -18280 bp	F	GTT GAC GTA CAG GGT GGA CTG
		R	GGA AGT TGT CTT CAC GCC TGA
	ETS	F	GAG TGC GGC TCG TCG CCT AC

Southern blotting and qPCR for rDNA copy number analysis	1601 – 2089 bp	R	TCC CAC CGC CAC AGA CAC GA

Supplementary Table 4: Antibodies

Proteins	Origin	Antibody details	Application
**Primary antibodies**			
γH2AX (S139)	Rabbit	Abcam (ab81299)	IF: 1:200
UBF1/2	Rabbit	a gift from Professor Larry Rothblum, University of Oklahoma Health Sciences Centre, Oklahoma City, USA	qChIP: 8 μl sera /sample; WB: 1/ 5,000
POLR1A (A194)	Rabbit	a gift from Professor Larry Rothblum, University of Oklahoma Health Sciences Centre, Oklahoma City, USA	qChIP: 8 μl sera /sample
**Secondary antibodies**			
Alexa Fluor 488 Goat-α-rabbit IgG	Donkey	Invitrogen (A11008)	IF: 1:2,000
Alexa Fluor 594 Goat-α-rabbit IgG	Donkey	Invitrogen (A11012)	IF-FISH: 1:2,000
